# Smart Home Product Layout Design Method Based on Real-Number Coding Genetic Algorithm

**DOI:** 10.1155/2022/1523330

**Published:** 2022-07-06

**Authors:** Nan Jiang

**Affiliations:** Department of Space Design, Kookmin University, Seoul 02707, Republic of Korea

## Abstract

Aiming at the problems of poor layout design efficiency of smart home products and low rationality of layout planning, a layout design method for smart home products based on a real-number coding genetic algorithm is proposed. The principles of smart home product layout design are analyzed and the smart home system architecture based on the Internet of Things is designed. Divide the combination of smart home product layout space according to spatial function, extract the visual features of smart home product layout, build a smart home product layout optimization model based on the two constraints of the total area and individual area of smart home product spatial layout, and design a real-coded genetic algorithm. The model is solved to improve the global convergence of the algorithm, and the optimization method for the layout of smart home products is obtained. The experimental results show that the layout design method of smart home products based on a real-number coding genetic algorithm can accurately extract the layout features of smart home products and accurately classify the number of pairs of homes. The efficiency of layout design and rationality of layout planning of smart home products are better, which reflects the effectiveness of this method.

## 1. Introduction

In the era of rapid development of information and economy, people's requirements for the home environment are constantly improving. Especially in the design of home furnishing and furnishings, the transformation from function-based to more beautiful and practical value, if the hard decoration is the “skeleton” of the building, the soft furnishing and furnishings of the home are the “flesh and blood” essence of its space part. The embodiment of modern home furnishings in the interior space presents a variety of designs with different styles [1]. The smart home is based on housing as a platform, using integrated wiring technology, communication technology, automatic control technology, and other related technologies to analyze the communication mode between the home and outside. The extensive use of smart home can effectively improve the quality of life. Multifunctional furniture can achieve different functions in a narrow range and achieve the purpose of flexible transformation and space-saving. Ensure the accuracy, immediacy, and stability of communication between wireless home systems, better meet the multilevel management needs of smart home monitoring, remote control, communication, etc., and make more contributions to the improvement of residential intelligence [2]. With the improvement of living standards, people put forward higher requirements for the quality of home furnishing. For people with no experience in home design, it is usually a matter of trial and error to actually move the furniture around until you are satisfied. However, this restricts the user's free choice and combination of furniture, and the obtained results often fail to meet the functional and aesthetic requirements [3]. Therefore, studying the layout design method of smart home products has become an important research content in the current related research fields.

Reference [4] discusses the active interaction design strategy of conversational agents in smart home scenarios, discusses the characteristics of initiative from the perspective of decision-making rights and communication methods, and builds four active styles (straight advisor, blunt advice) on this basis: decision maker, euphemistic suggester, euphemistic decision maker. Through the “Wizard of Oz” experimental method to explore the influence of a user's gender, age, experience, and living status on active style preference, the optimization scheme of active interaction design of conversational agents in smart home scenarios is obtained. However, this method is mainly aimed at the interactive design of conversational agents in smart home products. The rationality of the layout of different types of smart home products needs to be further analyzed. Reference [5] designed a smart home system based on 2.4 G and ZigBee. The system consists of a cloud server, gateway node, 2.4 G remote control node, and ZigBee sensor network. The gateway uploads the data collected by the node to the cloud server and mobile App display through Wi-Fi, realizes the remote control of each ZigBee node through the mobile App or 2.4 G remote control module, and realizes functions such as security, environmental monitoring, remote control switch, and the intelligent lighting adjustment. However, this method is mainly aimed at the communication deployment of smart homes without an in-depth analysis of the communication capabilities under different smart home product layouts. At the same time, it is to test its performance according to different layout schemes. Therefore, it can be used as a reference in this paper when it is applied to the layout of smart home products.

In view of the above problems, this paper proposes a layout design method for smart home products based on a real-number coding genetic algorithm. Analyzed the principles of smart home product layout design and designed the smart home system architecture based on the Internet of Things. The combined distribution of the layout space of smart home products is studied, and the total area and individual area of the smart home product space layout are used as constraints to build a smart home product layout optimization model. On this basis, this paper starts with the real-number coding, using the characteristic that the sum of two pairs of chromosomes before and after the hybridization of coding chromosomes remains unchanged, and the maximum value of the offspring is the value taken by the parent, and the minimum value is the value taken by the parent, through the real-number coding genetic algorithm to solve the model, the smart home product layout optimization method is obtained quickly and accurately.

## 2. Materials and Methods

### 2.1. Layout Design of Smart Home Products

#### 2.1.1. Characteristics and Principles of Smart Home Product Layout Design

*(1) Features of Smart Home Product Layout Design*. The layout and design of a smart home are an important part of current creative life. Provide residents with a small-area living environment, maximize the use of resource space, and gradually accelerate the various needs of urban development. The multifunctional smart home system combines automatic control technology, sensor technology, network communication technology, and embedded technology so that users can access and manage home equipment through wireless network technology. Real-time monitoring of indoor equipment, centralized management of access control settings, and improvement of various alarm functions are effective ways to ensure the safe operation of home equipment [6]. The system of a multifunctional smart home can be subdivided into two levels: hardware and software. The hardware includes servers and home equipment, and the software is composed of various embedded software and mobile phone application software. Smart homes can carry out market innovation and planning and express their own attributes in the natural environment and humanistic characteristics. In fact, the design and architecture of a smart home have the following characteristics:

1. The important features of the all-around living environment can make the living environment within a small area have complete living functions. In daily life, all things can be integrated into the same environment, and the space structure can be fully utilized so that a small area of space can play infinite possibilities.
2. Culture is also an important part of the living environment. According to the different living environments of people, the customs, concepts, and habits show differences. The structure and design of a smart home should meet the basic requirements of cultural characteristics in the living environment and actively absorb the excellent traditional culture. From the perspective of customs, social values, etc., the living environment reflects the localization characteristics.
3. Give full play to the convenient features of the living environment. In the process of smart home product layout planning, a modern living environment is designed according to the current situation. According to specific problems, we propose corresponding plans, pay attention to the interdependence between the living environment and occupants, and gradually meet the needs of occupants for social resources. The layout design of smart home products should meet the actual needs and gradually reduce the waste of resources. For the vast majority of occupants to investigate and analyze, and finally get the most scientific design scheme [7].

*(2) Smart Home Product Layout Design Principles*. The location and rational planning of the residential area should pay attention to the harmonious connection between the residential area and the level of economic income and design an optimal smart home product structure. The following principles should be followed in the design:

1. The principle of integrity
2. The rational planning of the living environment must pay attention to and take into account the overall connection of the living environment. Design and rational planning are closely related projects, and the scheme cannot be designed from an isolated perspective. The needs of the occupants are the theme of this design. The design and structure of a smart home must first pay attention to the overall environment, and then the pattern, modeling style, material selection, etc., must respect the aesthetic foundation, rationally plan the overall design of the living environment, and further complete the visual aesthetics and aesthetics, the laws of harmony and unity that life requires.
3. The principle of comfort
4. The structure and design of a smart home are important principles to be followed in the performance principle of the living environment. This principle can be applied in smart home planning. The fresh and convenient living environment can meet the material and spiritual requirements of residents in the new era. To meet the basic living convenience, as well as reasonable activity space, we try our best to meet the living needs of residents and improve their aesthetic level of residents. Let every resident have a healthy, comfortable, beautiful, and convenient living environment.
5. The principle of function as the main
6. The economic development needs of different households are different, and the design and structure should be analyzed in combination with the actual situation. Adopt a functional design method to promote the efficient and stable development of the intelligent design. For the smart home design concept, the geographical conditions of the residential area should be fully considered. Choose a design scheme with appreciative and practical value and discuss the architecture and design of smart homes in combination with environmental and economic conditions [8]. Reduce costs as much as possible, formulate a basic design scheme based on creative features, and make full use of the advantages of environmental space to design products and structures with more creative features.

#### 2.1.2. Architecture Design of Smart Home System Based on Internet of Things

The Internet of Things can generally be divided into five layers from bottom to top: perception control layer, access layer, Internet layer, service management layer, and application layer.

The main function of the perception control layer is to “perceive” the environmental parameters and the working parameters of the smart home products and to change the working state of the smart home products as needed. These devices all have ZigBee wireless interfaces [9, 10] to communicate with IoT gateways located at the access layer.

The main device in the access layer is the IoT gateway, which is mainly responsible for connecting many terminals in the perception and control layer to the Internet. On the one hand, it communicates with the terminal of the perception control layer through ZigBee or other interfaces and forwards the data sent by the terminal to the server or forwards the remote control command of the server to the terminal, and on the other hand, it has Ethernet, Wi-Fi, or GPRS, etc. This communication interface can access the local area network of the cell to communicate with the remote server [11].

The main devices in the Internet layer are those general-purpose network devices that are responsible for associating the IoT network with the local area network in the cell and then accessing the Internet or directly accessing the computer network of the network operator. The former can be the switch in the user's home and the network equipment such as the switch or router in the community, and the latter can be the ADSL modem, Cable Modem, wireless router, fiber router, and other equipment. Of course, both include many central office equipment of the operator. The service management layer mainly includes the application server, Web server, and database server.

The application server is responsible for regular communication with each IoT gateway, obtains the data of the sensing and control layer devices through the gateway, and saves it to the database server in time, while the web server [12, 13] is responsible for publishing the data to the Internet for users to view related information remotely through the browser.

The application layer mainly includes various computing devices such as desktop computers, laptop computers, tablet computers, and smartphones. Its main function is to provide users with a man-machine interface that can interact with the system remotely through a web browser or client software.

**Figure 1:**
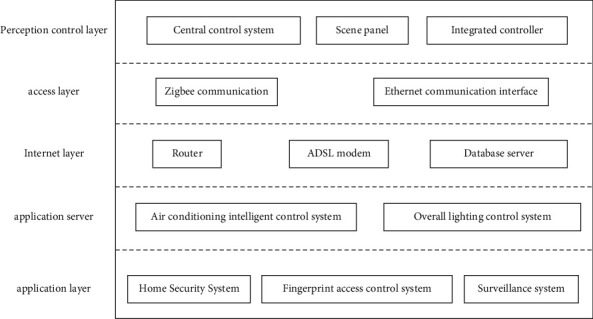
The architecture of the smart home system based on the Internet of Things.

In summary, the architecture of the smart home system based on the Internet of Things is shown in Figure 1.

The Internet of Things gateway plays an important role in the whole smart home Internet of Things, and it is a bridge connecting various terminal devices and servers. The gateway communicates with the terminal device through the ZigBee interface or serial port to obtain data and forward the data to the server through the Ethernet or Wi-Fi, or GPRS interface. The design of the gateway adopts the idea of modularization, and it is subdivided into three products: an Ethernet interface, a Wi-Fi interface, and a GPRS interface, according to the common Internet access methods in the family. Users can choose suitable products according to their own conditions.

Because the IoT gateway needs to complete many tasks, in order to better coordinate the operation of each task, an embedded operating system, C/OS-II, is used as its software platform. After the introduction of C/OS-II, application design became very simple. The application is divided into seven user tasks and one system task according to what the gateway should do. The priority is assigned according to the importance of the task and whether it has hard real-time performance. The lower the priority value, the higher the priority of the task. The main function of several communication tasks is to process the data received or sent by their corresponding interface chips. The processing of these data is strictly time-limited. If it is not processed in time, it may be corrupted by the data in the next data packet. So, these communication tasks have priorities over other tasks. Among these communication tasks, the Ethernet communication task has the highest priority because the communication rate of Ethernet is the fastest, and the length of the data packet is the longest [14].

One of the main features of the smart home Internet of Things is the ability to connect various smart home products in the house to the Internet, but it is not enough to be able to communicate with devices through the Internet, and the interaction between users and devices must also be realized on the Internet. The application server has stored the device data in the database, and the task of the web server is to display the data on the Internet so that users can view the environmental information and device status in the room through a web browser anytime, anywhere, and can also remotely control the operation of the device state.

### 2.2. Design Method of Smart Home Product Layout Based on Real-Number Coding Genetic Algorithm

According to the characteristics and principles of smart home product layout design, on the basis of clarifying the smart home system architecture under the application of Internet of Things technology and based on a real-coded genetic algorithm, the smart home product layout design is completed in three parts: the combination of smart home product layout space, the construction of the smart home product layout planning model, and the model solution based on a real-coded genetic algorithm.

#### 2.2.1. Combination of Smart Home Product Layout Space

According to the function of the space, first locate the overall style, matching colors, and furniture styles; after these are determined, the ceiling, walls, and floors of each interior space interface are decorated and designed. In terms of interior space, it is best to combine soft decoration and hard decoration and use furniture to combine, divide, and contrast the layout and design of the space.

*(1) Combination of Spaces*. Combined interior space is overall planning based on the functional requirements of the building, and the orderly organization from the whole to the single divided space is manifested in the sorting of the size of the space and the household objects that can be accommodated, forming a gap between the indoor space and the home furnishings. The organic connection of the interior space achieves coordination and unity in function and aesthetics, which is also the basis of interior space design. For example, the living room is a space composed of sofa seats, coffee tables, carpets, cabinets, lamps, TV sets, TV cabinets, curtains, and other furniture, providing people with functional areas for leisure, meeting, gathering, and audio-visual. The bedroom can be composed of beds, bedside tables, wardrobes, TVs, dressers, lamps, etc., and mainly provides space for people to rest. There are many forms of combining and expressing various indoor functional spaces, such as axis-symmetrical, centralized combined, radial, and so on.

*(2) Division of Space*. In addition to a certain connection, each space also has its own independence, which is mainly reflected by way of separation. The separation and connection of space is the separation and connection of space in the vertical and horizontal directions. A good separation should be organized in an orderly manner, with appropriate virtual and real and self-contained systems. This is of great significance to the effect of the entire space design and can present a unique design style. The characteristics of the home furnishing are as follows: Partial separation. Partial separation is the use of furniture such as screens and higher cabinets to block the space, but it does not completely close the space so that the lighting of the space has a breathable feeling which is common in traditional design layouts. Symbolic separation. Symbolic separation is the use of glass, greenery, color, material, height difference, overhangs, and other factors to separate the space, which reflects the effect of continuous separation in space division. This division method is mostly reflected in modern home design. Flexible separation. Elastic separation is the use of folding, lifting, and other activities, such as partitions and curtains, and other furniture, to separate the space, which can be closed or moved according to the requirements of use, and the space can be divided and closed, and the space can be freely stretched. Virtual separation. Virtual separation is the feeling of two spaces formed by different placement or independent design of furniture, carpets, and ceilings in the same space, and the two different functional spaces formed are independent and shared. This design method is usually suitable for small or open layouts.

*(3) Contrast of Space*. The relationship between furniture and interior space should conform to ergonomics, and changes in its size and color also directly affect people's psychological space [15, 16]. The contrast of the space can also be reasonably adjusted by the height, size, and color of the home furnishings so that the space has a well-proportioned sense of rhythm and forms a rhythmic beauty. Designers often use the proportions of furniture and furnishings to integrate them into the design ideas of interior spaces.

#### 2.2.2. Construction of Smart Home Product Layout Planning Model

The smart home product layout approach in this paper allows users to define orientation and distance constraints between furniture. In order to reduce the interaction, the system defaults that all furniture has distance and angle constraints with the room. Therefore, after the user interaction ends, a directed graph of the furniture relationship will be obtained. The nodes of the graph are composed of (1) room and (all) furniture; the edges of the graph indicate that the nodes have a constraint relationship, and the direction indicates the primary and secondary relationship when the user selects, that is, when selecting a furniture pair; the main furniture is selected first, and then the secondary furniture and the directed edge points from the primary furniture to the secondary furniture. Although it is assumed that the user has a certain basic common sense of interior decoration, it does not require the user to make a detailed design of the hierarchical relationship but allows the user to input any furniture pair constraints, so there will be cycles, bidirectional edges, etc., in the furniture relationship directed graph situation.

In order to get a hierarchical tree, it is necessary to delete all the incoming edges of the room node and make it the root of the hierarchical tree; secondly, check each furniture node so that it has only one incoming edge.

A two-step strategy using a simple size comparison:

  Strategy 1: Check all adjacent node pairs; if there are two-way edges, delete a directed edge from the small furniture to the large furniture; if there is only one one-way edge and it is from the small furniture to the large furniture, then flip the direction of the edge.
  Strategy 2: Check the neighbors of all incoming edges of each node and keep the incoming edge of the smallest adjacent node.

Establish a resolution multidimensional space block image fusion model for the extraction of visual features of smart home product layout, using block pixel matching method to achieve deep evolutionary learning of visual features of smart home product layout, combined with high-resolution information fusion detection through linear filtering. The pixel reorganization of the edge area realizes the extraction [17, 18] and segmentation of the visual features of the smart home product layout. The segmentation formula is as follows:

(1) $$\begin{array}{c} f=q+\frac{x}{v}. \end{array}$$

Among them, *q* is the resolution of visual feature extraction of smart home product layout, *x* is the block time interval parameter, and *v* is the joint information entropy of feature extraction.

Through the method of two-dimensional parameter fitting, the fitting coefficient of the multidimensional spatial block image for the visual feature extraction of the smart home product layout is obtained as *j*, and the multilevel visual feature extraction of the smart home product layout is carried out in a single-pixel value distribution area *i*. By dividing into blocks, the multilevel feature information of the indoor space layout can be obtained as follows:

(2) $$\begin{array}{c} K=i\left(f\times c\right)+j\left(f\times b\right). \end{array}$$

According to the multiscale machine learning results, the visual image reorganization of smart home product layout is carried out, and the fuzziness of smart home product layout visual feature extraction is represented by (*c*, *b*, *m*, *r*) quadruple, *y*^*e*^, *y*^*r*^ is the entity set for smart home product layout visual feature extraction. Combined with the analysis results of the constraint parameters of the restored image [19], the background value fusion of the visual image of the smart home product layout is realized, and the output is as follows:

(3) $$\begin{array}{c} C=\frac{K\left(y^{e}+y^{r}\right)}{f}+r+\frac{x}{v}. \end{array}$$

Select the nearest neighbor function *xy* group, and the ambiguity distribution set for visual feature detection of smart home product layout is established. From the perspective of feature reuse in the middle layer, the boundary feature components of visual feature detection of smart home product layout are obtained as follows:

(4) $$\begin{array}{c} S=\frac{xy}{C}+r,\\ T=\frac{2\pi \left(g^{o}+g^{u}\right)}{S}. \end{array}$$

Among them, *g*^*o*^ and *g*^*u*^ are the resolution and information entropy of the visual feature extraction of smart home product layout [20], respectively.

To sum up, build a building interior space layout planning model.

*(1) Objective Function*. Considering the size of the building space, set the objective function of smart home product layout planning as follows:

(5) $$\begin{array}{c} f\left(x\right)=\max\sum_{k=1}^{K}c_{k}z_{k}. \end{array}$$

In the formula, *f*(*x*) describes the building interior space layout planning function; *c*_*k*_ represents the number of *k*-type smart home products in the building interior space; *z*_*k*_ represents the space ratio corresponding to *k*-type smart home products.

*(2) Constraints*. The total area constraints for the spatial layout of smart home products are described by the following:

(6) $$\begin{array}{c} A=\sum_{k=1}^{K}Z_{K}. \end{array}$$

In the formula, *A* represents the total area of the interior space of the building.

The individual area constraints of smart home products are described by the following formula:

(7) $$\begin{array}{c} A_{J}\leq z_{J}\leq m+\theta . \end{array}$$

In the formula, *A*_*J*_ represents the minimum demand area of smart home products in the building space; *m* represents the land area for smart home products; *θ* represents the planned land area for smart home products.

#### 2.2.3. Model Solving Based on Real-Coded Genetic Algorithm

**Figure 2:**
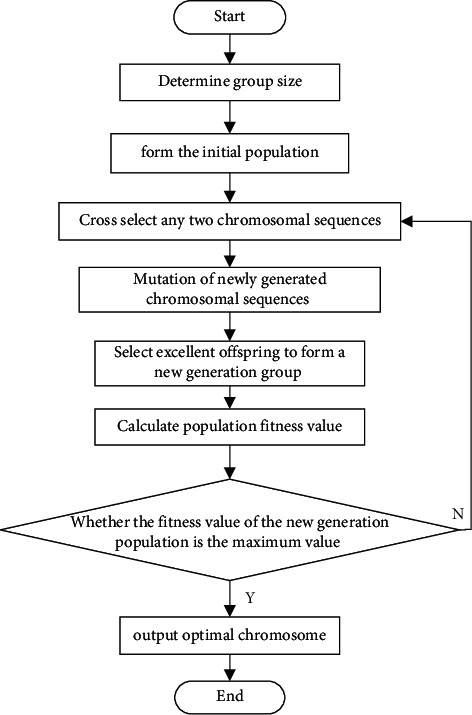
The solution flow of the real-coded genetic algorithm.

The smart home product layout design method based on real-number coding and real-number coding genetic algorithm adopts the real-number coding genetic algorithm [21, 22] to solve the smart home product layout model and realize the optimal design of the smart home product layout. The specific steps are as follows:

(1) Encoding in the form of 2 rows and *R* columns.
(2) Build fitness function *f*(*m*):
  (8) $$\begin{array}{c} f\left(m\right)=\left\{\begin{array}{cc} C_{\max}-f\left(m\right), & f\left(m\right)<C_{\max},\\ 0, & f\left(m\right)\geq C_{\max}. \end{array}\right. \end{array}$$
In the formula, *C*_max_ is a larger constant.
(3) Initial population: Most scholars randomly select the initial population, thinking that random selection can traverse all states, but the evolutionary generation of random selection increases, which increases the time required for solving and reduces the efficiency of the method. Based on real-number coding, the smart home product layout model construction method based on a genetic algorithm initializes the population through the following methods [23]:
  Step 1: Set a smaller number *M*, let *i* = 0, *f*_*i*_=*M*;
  Step 2: Calculate the fitness value *f*_*i*(*X*_*i*+1_)_ corresponding to the randomly generated chromosome *X*_*i*+1_, and calculate the average fitness value *f*_*i*_ corresponding to the first *i* − 1 chromosomes by the following formula:
    (9) $$\begin{array}{c} f_{i}=\frac{\sum_{i=0}^{i-1}f_{i}\left(X_{t}\right)}{f_{i\left(X_{1}\right)}}. \end{array}$$
  Step 3: Determine whether the population accepts individuals through the following process:
    (1) When *f*_*i*(*X*_*i*+1_)_ < *f*_*i*_, *i*+1 < *n*, then *X*={*X*_1_,…, *X*_*i*+1_}, *i*=*i*+1, and return to the previous step;
    (2) When *f*_*i*(*X*_*i*+1_)_ ≥ *f*_*i*_, *X*={*X*_1_,…, *X*_*i*_}, go back to the previous step;
    (3) When *f*_*i*(*X*_*i*+1_)_ < *f*_*i*_, *i*+1=*n*, when, *X*={*X*_1_,…, *X*_*i*+1_}, go to the next step.
  Step 4: End.
(4) Use the roulette selection method to realize the selection of strategies [24]. The specific steps are as follows:
  Step 1: Rank the fitness of individuals in the population in descending order to obtain the sequence {*X*_*N*1_,…, *X*_*Ni*_, *X*_*Nn*_} of the sorted chromosomes.
  Step 2: Use the probability sequence as the basis and basis in the roulette selection process, determine the real number *α*, obtain *n* numbers through the following, and use it as the basis in the selection probability process:
    (10) $$\begin{array}{c} p_{i}=\prod_{i=1}^{i}\alpha_{i}. \end{array}$$
  Step 3: Normalize the selection probability and calculate the selection probability of each individual by the following formula:
    (11) $$\begin{array}{c} p_{i}=\frac{p_{i}}{\sum_{i=1}^{n}p_{i}}. \end{array}$$
  Step 4: Calculate the cumulative probability *q*_*i*_ corresponding to the chromosome in the next generation population:
    (12) $$\begin{array}{c} q_{i}=\sum_{i=1}^{i}p_{i}. \end{array}$$
  Step 5: Select individuals in the population to enter the next generation. When *q*_*i*_ ≤ *r* ≤ *q*_*i*+1_, chromosome *X*_*Ni*_ can enter the next generation population.
(5) Crossover operation, the crossover operation is realized by the following formula:
  (13) $$\begin{array}{c} p_{c}=\frac{t\left(p_{\max}-p_{\min}\right)}{T}. \end{array}$$
In the formula, *p*_*c*_ represents the crossover probability; *p*_max_ and *p*_min_ represent the maximum crossover probability and the initial crossover probability, respectively.
(6) Mutation operation, in the process of group evolution, the mutation probability is adjusted and calculated by the following formula:
  (14) $$\begin{array}{c} p_{m}=\frac{p_{m1}+p_{m2}}{2}. \end{array}$$
(7) Termination condition, the construction method of smart home product layout model, based on real-number coding genetic algorithm adopts the double termination convergence criterion [25], that is, continuous unimproved maximum genetic algebra method. In the iterative process, if any of the above rules are met, the iteration will be stopped, and the optimal solution of the smart home product layout optimization model will be output to realize the optimal design of the indoor space layout. The real-coded genetic algorithm solution process is shown in Figure 2:

## 3. Results and Discussion

### 3.1. Experimental Parameter Settings

**Figure 3:**
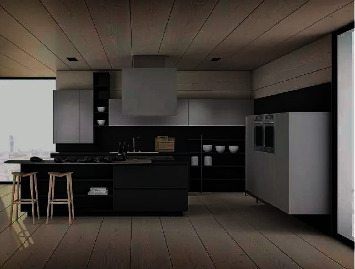
Visual sampling of smart home product layout.

In order to verify the effectiveness of the smart home product layout design method based on the real-number coding genetic algorithm, this paper downloads a large number of (to be counted) furniture models from the 3D model website and categorizes them according to the basic labels, including cabinets, chairs, tables, coffee tables, shelves, dining tables, TVs, etc., and manually consistent size and orientation of each furniture model. The feature extraction simulation of the smart home product layout is carried out, and the visual sampling of the smart home product layout is obtained, as shown in Figure 3.

The parameters of a real-coded genetic algorithm are as follows: the initial population size is 20; Maximum genetic algebra 50; The probability of crossing is 0.8; The window variation probability is 0.04 and 0.8; When the appropriate value of the mutated individual is not better than that of the premutated individual, the probability of replacing the premutated individual is 0.05.

### 3.2. Experimental Results and Analysis

The performance of different methods for extracting layout features of smart home products is tested, and the comparison results are shown in Table 1.

Analysis of the simulation results shows that the method proposed in this paper has a high degree of recognition and a better ability to distinguish the layout features of smart home products, which improves the display and expression capabilities of smart home product layouts.

This paper selects 5 scene instances for comparative analysis, which correspond to the living room, study, bedroom, writing, and dining room, and 5 kinds of home or workplace. The active interaction design method of the smart home dialog agent in reference [4] and the design method of the IoT smart home control system based on 2.4 G and zigzag in reference [5] are used as experimental comparison methods, the optimization effect of smart home product layout design is tested, and the comparison results of smart home product layout optimization design are shown in Table 2.

From the analysis of Table 2, it can be seen that due to the problem of constraint conflict, the solution space of the problem is complicated, which makes it difficult for the reference method to obtain the optimal solution in a limited time. The complexity of the furniture arrangement problem is not only related to the number of furniture but also related to the logarithm of the paired relationship between the furniture. The resulting problem is more complicated. Therefore, with more furniture, more crowded rooms, and more complex pairing relationships situation, it will be more difficult to solve. Therefore, the real-number coding genetic algorithm proposed in this paper decomposes the constraint conflict of the paired relationship so that the paired relationship between the furniture has a clear hierarchy. Local optimization and then global optimization can decompose complex problems into simple ones. Therefore, in the optimization design of the home product layout, the paired quantity of the home is accurately classified.

**Figure 4:**
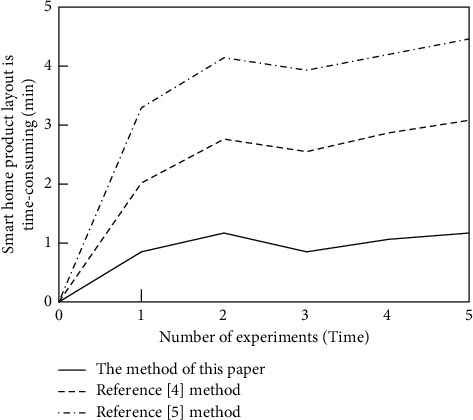
Smart home product layout design efficiency test results.

Taking the smart home product layout time as an indicator, test the smart home product layout design efficiency of this method, reference [4] method, and reference [5] method. During the test, taking the visual sampling of the smart home product layout shown in Figure 3 as the standard, three methods are used to test the smart home product layout of a new room. The test is divided into five groups. The rooms in each group are different in size. The specific test results are shown in Figure 4.

From the data in Figure 4, it can be seen that compared with the method in reference [4] and the method in reference [5], the method in this paper takes a shorter time to design the layout of smart home products and the average time is 1 min, because the method in this paper is used in smart home products. Based on the layout optimization model, the layout design of smart home products is realized by combining the idea of constraint solving and a step-by-step refinement strategy, which shortens the time required for the layout design of smart home products and improves the efficiency of layout design of smart home products.

**Figure 5:**
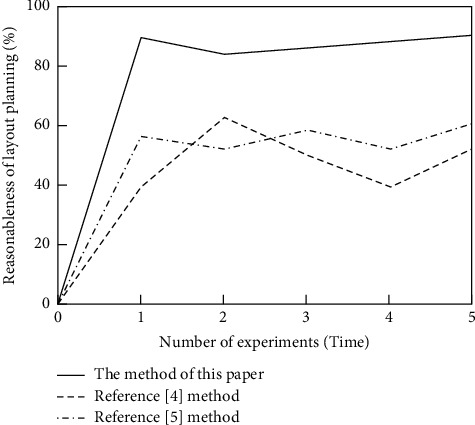
Layout planning rationality test results.

In order to further verify the effectiveness of this method, this method, reference [4] method, and reference [5] method are used to plan the space of smart home products, and the rationality of layout planning of different methods is compared. During the test, take the average of 200 users' satisfaction with the layout of five groups of different rooms as a result of statistics, and the test results are shown in Figure 5.

Analysis of Figure 5 shows that the rationality of the spatial layout of smart home products after the method in this paper is more than 80%, and the average is 89%, which is much higher than that of the method in reference [4] and the method in reference [5] after the layout planning. The rationality of the space layout of smart home products. The method in this paper establishes a smart home product layout optimization model on the basis of extracting the characteristics of smart home products and uses a real-number coding genetic algorithm to solve it, which improves the rationality of layout planning.

## 4. Conclusions

There are many kinds of smart home products which are prone to constraint conflicts in the layout process. This paper presents a real-coded genetic algorithm-based smart home product layout design method. This paper establishes a hierarchical tree based on the prior pair relationship between furniture to degrade the constraint conflicts and builds a smart home based on the two constraints of the total area and individual area of the smart home product space layout; product layout optimization model, which also reduces the complexity of the search problem and improves the speed of the search. In addition, in order to further improve the solution efficiency, this paper introduces the real-number coding genetic algorithm to optimize the solution. The real-coded genetic algorithm has a faster convergence rate, and since it starts searching from multiple initial values at the same time, it also improves the quality of the search. Finally, the experiment is carried out with five scenarios. The experimental results show that the average time of smart home product layout design is 1 min, and the average value of layout planning rationality is 89%. The results show the effectiveness of this method. The scene after the application of this method meets the needs of functionality and aesthetics.

## Figures and Tables

**Table 1:** Feature extraction performance of smart home product layout.

Testing frequency	Feature extraction accuracy (%)	Spatial resolution [dpi]
10	94.6000	4.7684
20	97.3200	4.3474
30	93.9600	4.4000
40	98.2800	4.4947
50	92.6800	4.5579
60	93.3200	4.3158
70	90.9200	4.6737
80	92.2000	4.5474
90	93.8000	4.0526
100	90.7600	4.4000
110	89.4800	4.5158
120	88.5200	4.4000

**Table 2:** Comparison results of smart home product layout optimization design.

Scenes	Number of homes	Pair relationship		
		The method of this paper	Reference [4] method	Reference [5] method
Living room	16	Sofa and TV	Sofa and TV	Sofa and coffee table
		Sofa and coffee table	—	Coffee table and TV
		Coffee table and TV	—	—

Study	20	Table and chair	Coffee table and shelf	Coffee table and stool
		Coffee table and stool	—	Coffee table and shelf
		Coffee table and shelf	—	—

Bedroom	14	Bed and nightstand	Bed and nightstand	Bed and nightstand
		Bed and TV cabinet	—	Bed and TV cabinet
		Dresser and stool	—	—

Office	28	Desk and chair	Work desk and work desk	Desk and chair
		Work desk and work desk	—	—

Dining room	63	Dining table and chairs	—	Dining table and chairs

## Data Availability

The dataset can be obtained from the corresponding author upon request.
